# Novel strategies to mimic transmembrane tumor necrosis factor-dependent activation of tumor necrosis factor receptor 2

**DOI:** 10.1038/s41598-017-06993-4

**Published:** 2017-07-26

**Authors:** Roman Fischer, Jessica Marsal, Cristiano Guttà, Stephan A. Eisler, Nathalie Peters, John R. Bethea, Klaus Pfizenmaier, Roland E. Kontermann

**Affiliations:** 1 Institute of Cell Biology and Immunology, University of Stuttgart, Allmandring 31, 70569 Stuttgart, Germany; 2 Department of Biology, Drexel University, 3245 Chestnut Street, Philadelphia, PA 19104 USA; 3 Stuttgart Research Center Systems Biology, Nobelstraße 15, University of Stuttgart, Stuttgart, Germany

## Abstract

Tumor necrosis factor receptor 2 (TNFR2) is known to mediate immune suppression and tissue regeneration. Interestingly, the transmembrane form of tumor necrosis factor (tmTNF) is necessary to robustly activate TNFR2. To characterize the stoichiometry and composition of tmTNF during TNFR2 activation, we constructed differently oligomerized single chain TNF ligands (scTNF) comprised of three TNF homology domain (THD) protomers that mimic tmTNF. Using a variety of cellular and *in vivo* assays, we can show that higher oligomerization of the scTNF trimers results in more efficient TNF/TNFR2 clustering and subsequent signal transduction. Importantly, the three-dimensional orientation of the scTNF trimers impacts the bioactivity of the oligomerized scTNF ligands. Our data unravel the organization of tmTNF-mimetic scTNF ligands capable of robustly activating TNFR2 and introduce novel TNFR2 agonists that hold promise as therapeutics to treat a variety of diseases.

## Introduction

The tumor necrosis factor (TNF) superfamily is a family of different cytokines with various functions. Most ligands of the TNF family are synthesized as trimeric type II transmembrane proteins that can be released into a soluble form via proteolytic processing. The structural hallmark defining the TNF ligand family is the carboxy-terminal TNF homology domain (THD) which is composed of two stacked β-pleated sheets that adopt a conserved jellyroll-like tertiary fold^1–3^. This structural composition leads to the self-association of TNF monomers into trimers and is necessary for receptor binding^1, 3^. Due to the carboxy-terminal localization of the THD, both the transmembrane form as well as soluble TNF ligands assemble into trimers. However, the THD-mediated receptor interaction alone is not necessarily sufficient to activate receptor-associated intracellular signaling pathways. For several members of the TNF receptor superfamily, the initial formation of ligand receptor complexes is followed by secondary multimerization into supramolecular clusters^4–7^.

Despite their similar trimeric organization, membrane-bound and soluble TNF ligands can differ in their activity. This difference is specifically obvious for the name-giving family member TNF. TNF is synthesized as a trimeric transmembrane protein (tmTNF; 26 kDa) that can be released into soluble circulating TNF homotrimers (sTNF; 51 kDa) via cleavage of the ectodomain by TNFα-converting enzyme (TACE/ADAM17)^8^. Trimeric sTNF tends to irreversibly dissociate at subnanomolar concentrations, thereby losing its bioactivity^9^. Dissociation and thus inactivation can be prevented by connecting three TNF monomers with short intramolecular peptide linkers, resulting in covalently stabilized single-chain TNF trimers (scTNF)^10^.

TNF can bind two structurally distinct transmembrane receptors, TNF receptor (TNFR) 1 and TNFR2, which have marked differences in expression patterns, structure, signaling mechanisms and functions^11–13^.

Both tmTNF and sTNF can activate TNFR1 in the picomolar range, whereas TNFR2 is only robustly activated by tmTNF^14^. Different association/dissociation kinetics of the ligand/receptor complexes may contribute to the different TNFR activation capabilities of sTNF and tmTNF. Whereas sTNF has a remarkably high affinity for TNFR1 (K_d_ = 1.9 × 10^−11^ M), the affinity for TNFR2 is significantly lower (K_d_ = 4.2 × 10^−10^ M)^15^. The high affinity of sTNF for TNFR1 is mainly caused by stabilization of ligand/receptor complexes, while transient binding of sTNF to TNFR2 results in short-lived signal incompetent complexes^15, 16^.

A probable reason for the tmTNF-dependence for TNFR2 activation is the higher demand of TNFR2 for ligand-mediated crosslinking to allow signaling cluster formation^16, 17^. In this line, oligomerization of soluble ligand trimers, e.g. via antibody-mediated crosslinking, does not increase activation of TNFR1^18^, whereas secondary oligomerization of soluble TNF trimers converts this molecule into an active TNFR2 agonist^19, 20^.

To characterize the stoichiometry and composition of tmTNF necessary to efficiently activate TNFR2, we genetically engineered differently oligomerized TNFR2-selective scTNF ligands. The activity of these fusion proteins was then compared in regard to TNFR2 binding, TNF/TNFR2 complex formation and induction of specific cellular responses.

## Results

### Oligomerization of covalently stabilized scTNF dramatically improves affinity for TNFR2

**Figure 1:**
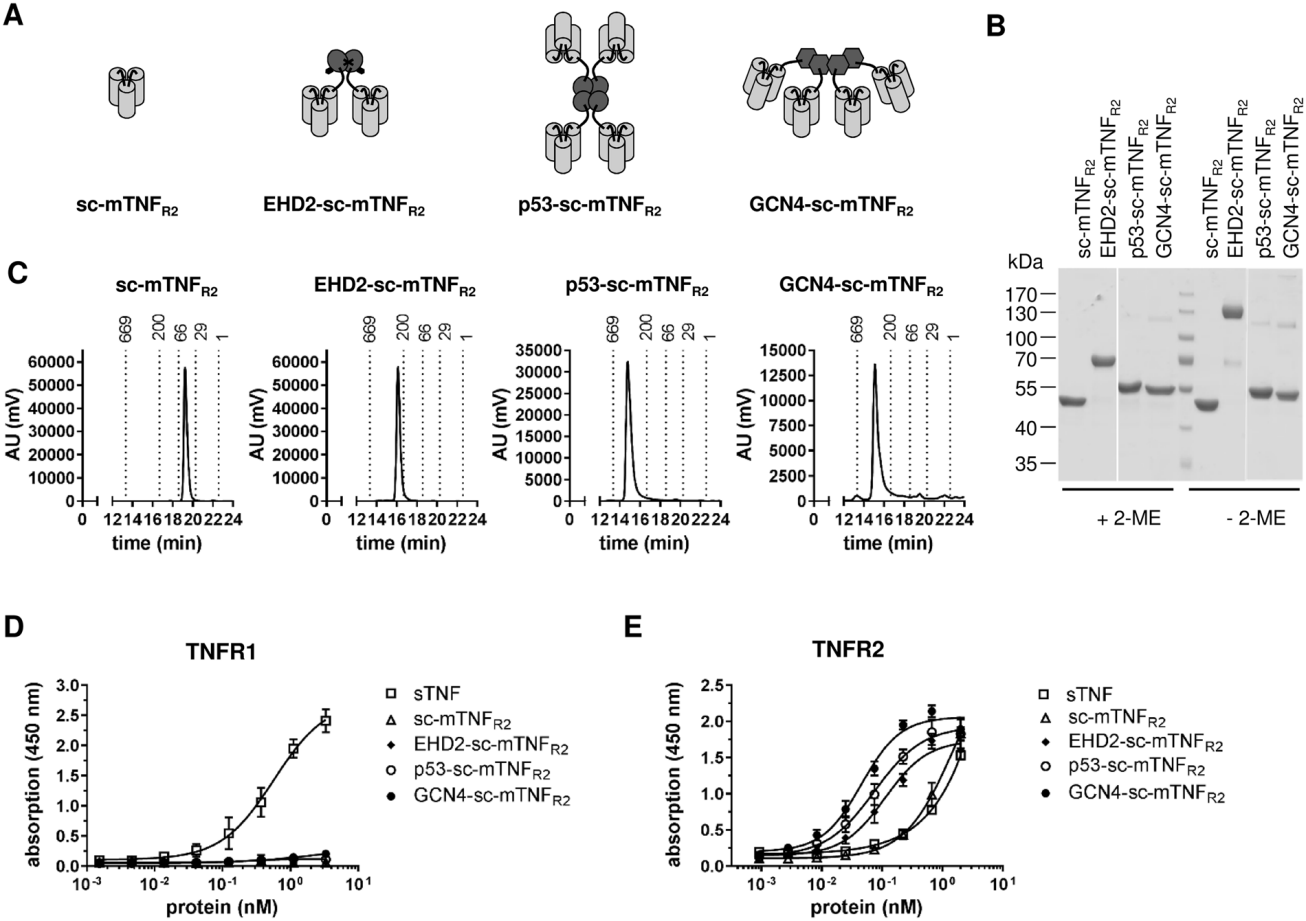
Characterization of oligomeric TNFR2 selective TNF muteins (**A**) Schematic representation and size exclusion chromatography analysis of the differently oligomerized TNF muteins sc-mTNF_R2_, EHD2-sc-mTNF_R2_, p53-sc-mTNF_R2_ and GCN4-sc-mTNF_R2_. (**B**) Coomassie staining of sc-mTNF_R2_, EHD2-sc-mTNF_R2_, p53-sc-mTNF_R2_ and GCN4-sc-mTNF_R2_. Purified TNF variants were analyzed by 8% SDS-PAGE under reducing (+2-ME) or non-reducing (−2-ME) conditions and stained with Coomassie. (**C**) Size exclusion chromatography analysis of the differently oligomerized TNF muteins sc-mTNF_R2_, EHD2-sc-mTNF_R2_, p53-sc-mTNF_R2_ and GCN4-sc-mTNF_R2_. (**D**,**E**) Binding of the TNF muteins to mouse (**D**) TNFR1 and (**E**) TNFR2 was analyzed by ELISA. Soluble recombinant mouse TNF (sTNF) was used as a positive control for binding to TNFR1 (n = 3 ± SEM).

Previously, we demonstrated that oligomerized, covalently stabilized scTNF_R2_ mimics tmTNF and efficiently activates TNFR2^19, 21^. We, therefore, fused a mouse TNFR2-specific (D221N/A223R) sc-mTNF (sc-mTNF_R2_) to different oligomerization domains, resulting in fusion proteins with different arrangements of the sc-mTNF_R2_ moieties (Fig. 1A, Fig. S1). For oligomerization, we applied the CH2 dimerization domain of IgE (EHD2^21, 22^), the tetramerization domain of p53 (aa 320–359)^23–25^ that exhibits an antiparallel arrangement of the domains (dimer of dimers) resulting in a tetrahedron-like 3D structure, and GCN4 (aa 249–281), a mutated helix from the yeast transcription factor GCN4^24, 26, 27^, with a parallel arrangement of the four coiled-coil domains^28^ (Fig. 1A). All fusion proteins were expressed in HEK293–6E cells, isolated by IMAC in a single step using a N-terminal his-tag present in the molecule and further purified by preparative size-exclusion chromatography (SEC). Purity was confirmed by SDS-PAGE and Coomassie staining (Fig. 1B). Under reducing conditions, the fusion proteins exhibited an apparent molecular mass of approximately 45 kDa (sc-mTNF_R2_), 70 kDa (EHD2-sc-mTNF_R2_) and around 55 kDa (p53-sc-mTNF_R2_ and GCN4-sc-mTNF_R2_), matching the calculated molecular mass of 50.2 kDa, 66.6 kDa, 59.7 kDa and 58.9 kDa for sc-mTNF_R2_, EHD2-sc-mTNF_R2_, p53-sc-mTNF_R2_ and GCN4-sc-mTNF_R2_, respectively. Under non-reducing conditions, an additional band of approximately 140 kDa (dimer) was observed for EHD2-sc-mTNF_R2_, indicating the disulfide-stabilized dimer formation via the EHD2 domain. Both p53-sc-mTNF_R2_ and GCN4-sc-mTNF_R2_ showed only partially oligomerized bands, which was expected since these oligomerization domains are not stabilized via covalent disulfide bonds. The oligomerization state of the fusion proteins was further confirmed by SEC (Fig. 1C). All fusion proteins eluted as a single major peak, indicating the integrity and high purity of the molecules. The apparent molecular masses correspond to the calculated molecular masses for monomers (50 kDa, sc-mTNF_R2_), dimers (200 kDa, EHD2-sc-mTNF_R2_) or tetramers (250 kDa, both p53-sc-mTNF_R2_ and GCN4-sc-mTNF_R2_).

Binding of the fusion proteins to TNFRs was analyzed by ELISA using immobilized mouse TNFR1-Fc and TNFR2-Fc fusion proteins. No binding to TNFR1 was observed at any concentration tested (Fig. 1D), verifying that even highly oligomerized TNF muteins have lost their affinity towards TNFR1. In contrast, all scTNF_R2_ muteins bound to TNFR2. Whereas the monomeric sc-mTNF_R2_ (EC_50_ value of 1.55 nM) only interacted with TNFR2 at high concentrations, similar to recombinant soluble TNF (sTNF), EC_50_ values of EHD2-sc-mTNF_R2_ (EC_50_ value of 0.11 nM), p53-sc-mTNF_R2_ (EC_50_ value of 0.074 nM) and GCN4-sc-mTNF_R2_ (EC_50_ value of 0.045 nM) were significantly lower, with GCN4-sc-mTNF_R2_ displaying the highest binding efficiency (Fig. 1E and Table 1).

**Table 1:** Overview of the EC^50^ values (nM) binding assay.

Protein	EC_50_ (nM)
sc-mTNF_R2_	1.55
EHD2-sc-mTNF_R2_	0.11
p53-sc-mTNF_R2_	0.074
GCN4-sc-mTNF_R2_	0.045

**Figure 2:**
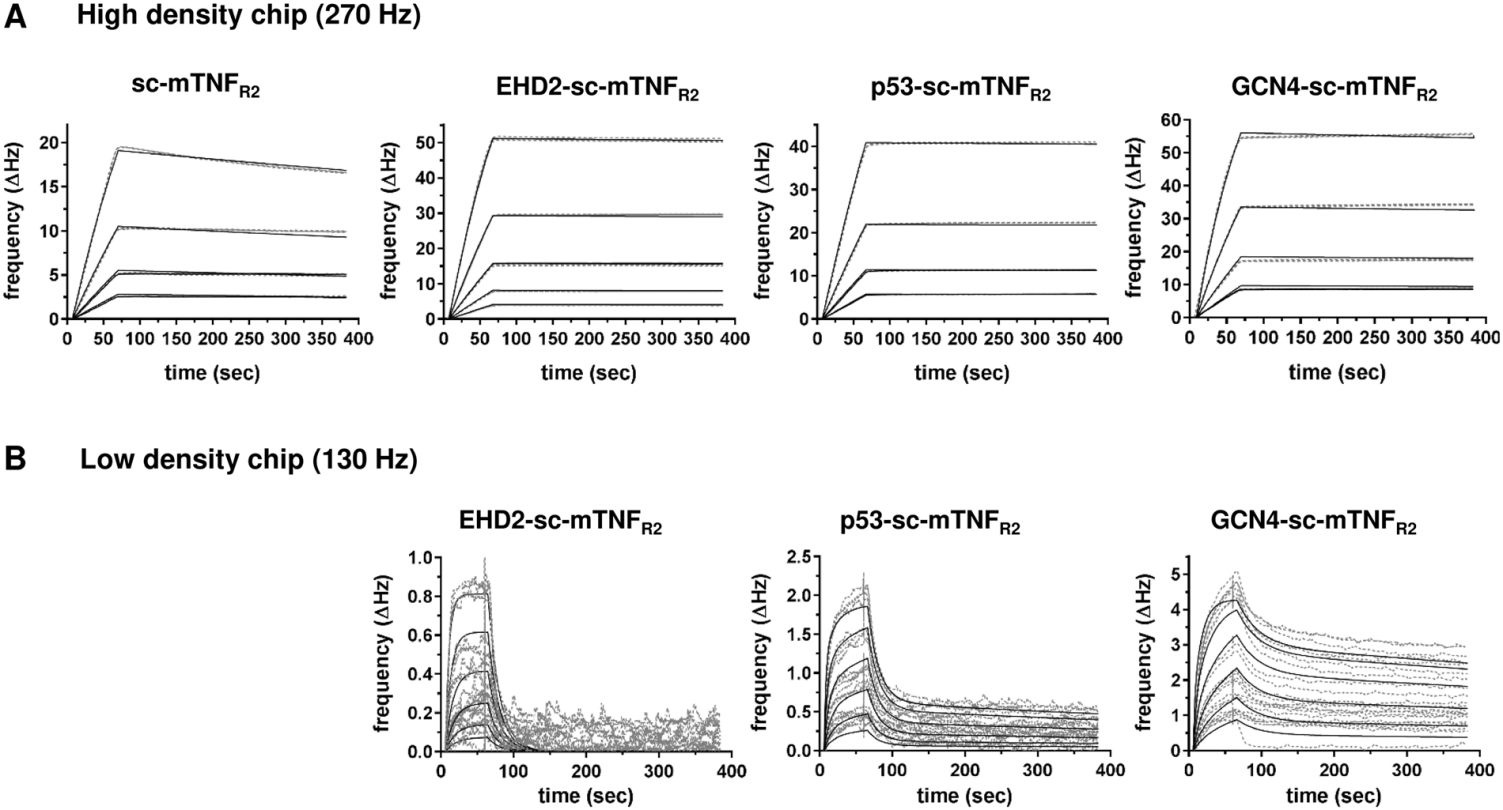
Dodecameric TNF muteins have higher affinity to TNFR2 Binding of oligomerized TNF muteins to mouse TNFR2 was analyzed by QCM at high (A; 270 Hz) and low (B; 130 Hz) density of immobilized mouse TNFR2-Fc. Oligomerized TNF muteins were analyzed at concentrations between 2–32 nM (**A**) or 8–256 nM (**B**) at 37 °C in triplicates for each concentration (dashed lines = data curves, solid lines = fitted curves).

Next, we used quartz crystal microbalance measurements to determine the affinity of the fusion proteins to mouse TNFR2-Fc. Using a high density chip (270 Hz) with saturated immobilized TNFR2, strong binding of sc-mTNF_R2_, EHD2-sc-mTNF_R2_, p53-sc-mTNF_R2_ and GCN4-sc-mTNF_R2_ to TNFR2 was observed, with K_d_ values between 61 to 44 pM (Fig. 2A and Table 2). In contrast, an around 10-times higher dissociation constants (K_d_) was determined for sc-mTNF_R2_. Minor differences were observed between EHD2-sc-mTNF_R2_, p53-sc-mTNF_R2_ and GCN4-sc-mTNF_R2_, with GCN4-sc-mTNF_R2_ showing the lowest off-rate. We, therefore, investigated, if differences between EHD2-sc-mTNF_R2_, p53-sc-mTNF_R2_ and GCN4-sc-mTNF_R2_ can be detected using a low density chip (130 Hz), with non-saturated immobilized TNFR2 (Fig. 2B and Table 2). Interestingly, at lower receptor density the curves for p53-sc-mTNF_R2_ and GCN4-sc-mTNF_R2_ followed a biphasic binding to immobilized TNFR2. Indeed, for both p53-sc-mTNF_R2_ and GCN4-sc-mTNF_R2_ lower K_d_ values were measured compared to EHD2-sc-mTNF_R2_, with GCN4-sc-mTNF_R2_ showing the most stable binding to the immobilized TNFR2.

**Table 2:** QCM affinity measurements of oligomeric TNF muteins.

	Protein	EHD2-sc-mTNF_R2_	p53-sc-mTNF_R2_	GCN4-sc-mTNF_R2_	sc-mTNF_R2_
high density	K_on_ (M^−1^ s^−1^)	6.07 × 10^5^	6.16 × 10^5^	1.67 × 10^6^	8.08 × 10^5^
	K_off_ (s^−1^)	3.74 × 10^−5^	2.69 × 10^−5^	8.77 × 10^−5^	3.99 × 10^−4^
	K_d_ (nM)	0.0615	0.0437	0.0527	0.4494
low density	k_on_1 [M^−1^ s^−1^]	5.33 × 10^5^	1.28 × 10^6^	2.16 × 10^6^	n/d
	K_off_1 [s^−1^]	6.3 × 10^−2^	6.51 × 10^−2^	3.47 × 10^−2^	n/d
	K_d_1 (nM)	118	50.9	16.1	n/d
	k_on_2 [M^−1^ s^−1^]	n/d	2.20 × 10^6^	3.45 × 10^6^	n/d
	K_off_2 [s^−1^]	n/d	8.76 × 10^−6^	5.97 × 10^−6^	n/d
	K_d_2 (nM)	n/d	3.97	1.73	n/d

**Figure 3:**
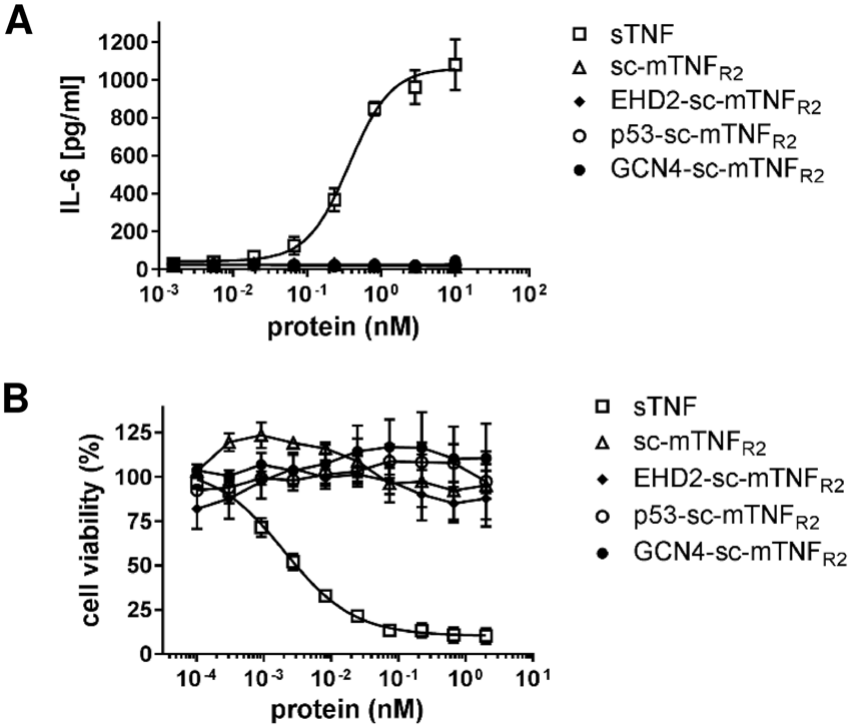
Dodecameric TNF muteins are still selective for TNFR2 (**A**) HeLa cells were stimulated with oligomerized TNF muteins for 24 hours. Soluble recombinant human TNF (sTNF) was used as a positive control for activation of TNFR1. Then supernatant was collected and analyzed for presence of IL-6 by ELISA (n = 3 ± SEM). (**B**) L929 cells were incubated with oligomerized TNF muteins for 24 hours. Soluble recombinant mouse TNF (sTNF) was used as a positive control for activation of TNFR1. Cell viability was measured using crystal violet staining (n = 3 ± SEM).

TNFR2 selectivity of the oligomerized TNF muteins was confirmed using HeLa and L929 cells. In contrast to wild-type TNF used as a positive control, none of the TNFR2-selective fusion proteins induced TNFR1-dependent IL-6 secretion in HeLa cells (Fig. 3A). Furthermore, none of the fusion proteins activated TNFR1-dependent cell death in L929 (Fig. 3B), verifying that also the higher oligomerized p53-sc-mTNF_R2_ and GCN4-sc-mTNF_R2_ had lost affinity for TNFR1 and the capacity to activate this receptor.

### Oligomerization of covalently stabilized scTNF is necessary for formation of higher order TNF/TNFR2 clusters

**Figure 4:**
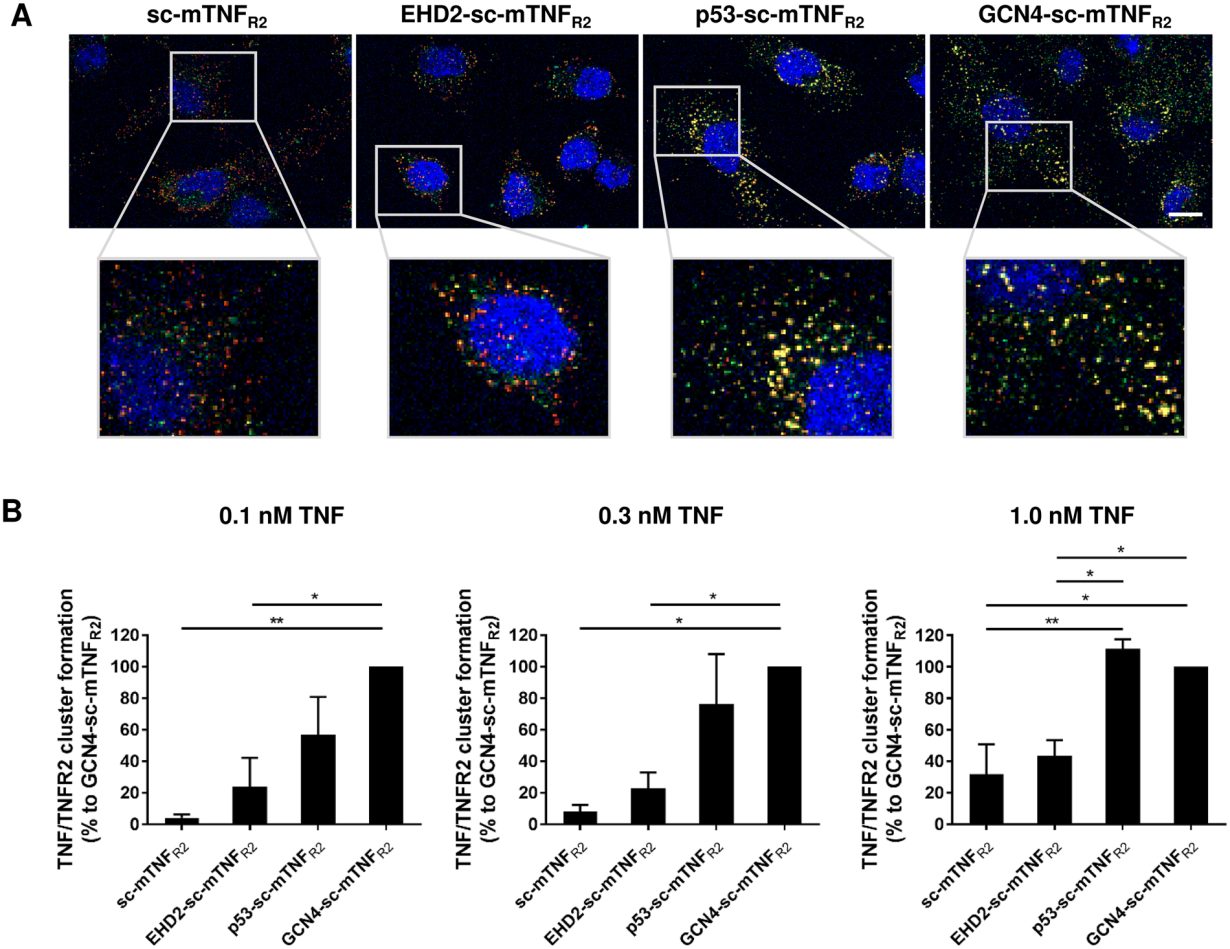
Dodecameric TNF muteins exert elevated TNF/TNFR2 cluster formation (**A**) BV-2 cells were incubated with the oligomerized TNF muteins (0.1, 0.3, 1.0 nM) for 15 minutes. Then cells were fixed and localization of TNFR2 (red) and oligomerized TNF muteins (green) was visualized by immunofluorescence microscopy. DAPI was used to counterstain cell nuclei. Fluorescence was analyzed using a Zeiss Axio Observer Spinning Disc microscope. Representative pictures for stimulation with 1 nM of the oligomerized TNF muteins are shown. Scale bar: 10 µm. (**B**) Quantification of TNF/TNFR2 clustering (n = 3 ± SEM).

Receptors of the TNF family are activated by ligand-mediated oligomerization^29^ and efficient signal initiation requires the formation of larger ligand/receptor complexes^16, 30^. We, therefore, investigated the differences between the oligomeric TNF muteins to induce TNF/TNFR2 signaling cluster formation. Mouse BV-2 microglia cells were incubated with different concentrations of the fusion proteins and TNF/TNFR2 cluster formation was visualized via immune fluorescence (Fig. 4A,B). At low concentrations (0.1 nM), GCN4-sc-mTNF_R2_ induced the highest amount of TNF/TNFR2 signaling complexes, indicated by yellow spots due to colocalization of TNF (green) and TNFR2 (red) signals. Around 50% less TNF/TNFR2 signaling complexes were induced with p53-sc-mTNF_R2_. Using a high concentration of 1 nM, similar efficient TNF/TNFR2 cluster formation (90–100%) was observed after incubation with p53-sc-mTNF_R2_ and GCN4-sc-mTNF_R2_. In contrast, both scTNF_R2_ and EHD2-sc-mTNF_R2_ were less capable of inducing TNF/TNFR2 cluster formation, with 30–40% clusters formed at the highest concentration. Notably, at low concentrations (0.1 nM), almost no cluster formation was observed with sc-mTNF_R2_, whereas EHD2-sc-mTNF_R2_ induced approximately 20% cluster formation.

### Oligomerization of covalently stabilized scTNF is necessary to robustly mimic tmTNF

**Figure 5:**
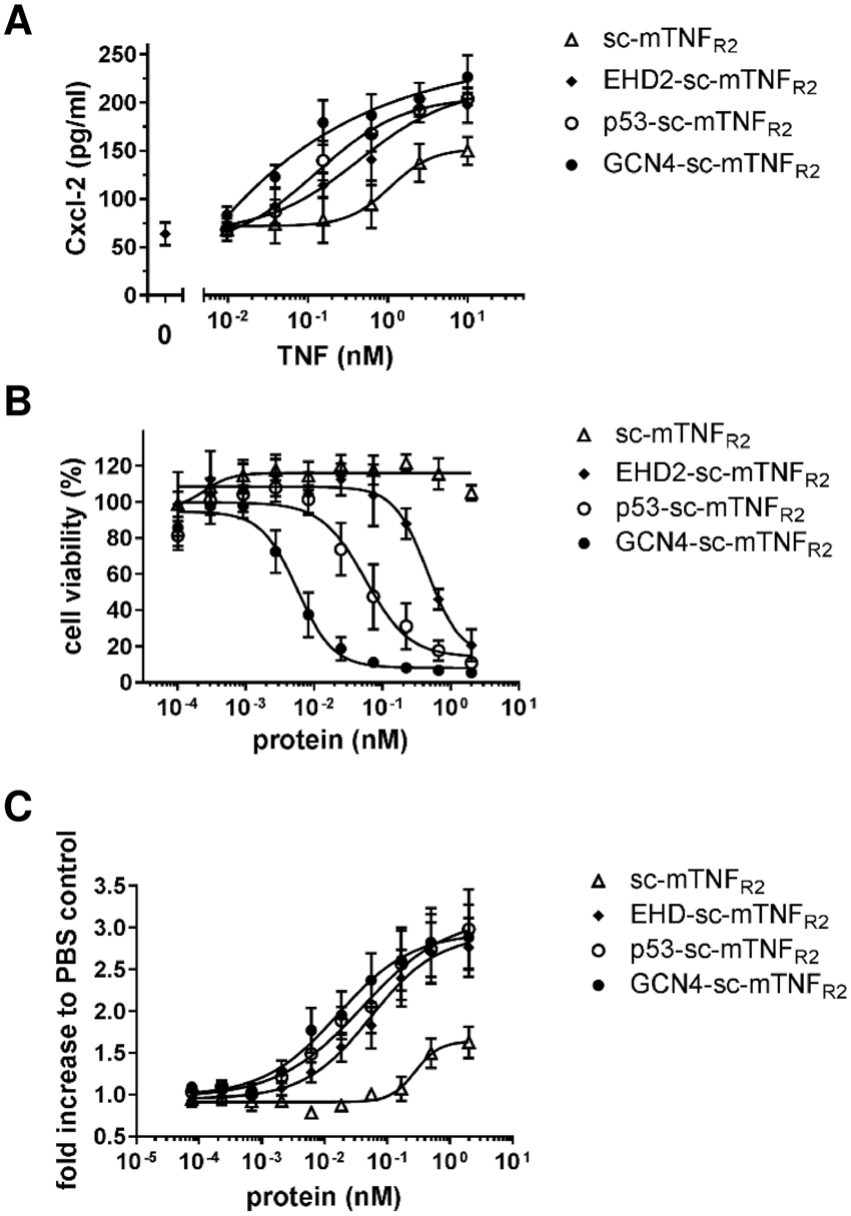
Dodecameric TNF muteins exert elevated bioactivity (**A**) BV-2 cells were incubated for 24 hours with the oligomerized TNF muteins. Then supernatant was harvested and analyzed for presence of secreted Cxcl-2 by ELISA (n = 3 ± SEM). (**B**) Thymocytes were incubated for 4 days in presence of the oligomerized TNF muteins. Then metabolic activity was quantified to determined relative cell numbers (n = 5 ± SEM). (**C**) Kym-1 cells were incubated for 24 hours with the oligomerized TNF muteins. Then cell viability was determined by crystal violet assay (n = 4 ± SEM).

We then investigated if the elevated binding capacity of the dodecavalent TNF oligomers is also converted to an increased bioactivity. First, we determined TNF_R2_-induced secretion of Cxcl-2 in BV-2 cells. Here, GCN4-sc-mTNF_R2_ (EC_50_ value of 32 pM) was 5-times more potent than p53-sc-mTNF_R2_ (EC_50_ value of 130 pM) (Fig. 5A and Table 3). Furthermore, an approximately 3.5-times elevated bioactivity of p53-sc-mTNF_R2_ compared to EHD2-sc-mTNF_R2_ (EC_50_ value of 438 pM) was observed, whereas sc-mTNF_R2_ induced the weakest Cxcl-2 secretion.

**Table 3:** Overview of the EC50 values (pM) bioactivity assays.

Protein	Cxcl-2 secretion (BV-2)	Cell death (Kym-1)	Proliferation (thymocytes)
sc-mTNF_R2_	1071	n/d	277
EHD2-sc-mTNF_R2_	438	453	55
p53-sc-mTNF_R2_	130	56	40
GCN4-sc-mTNF_R2_	44	5.7	16

Bioactivity of the fusions proteins was further tested using Kym-1 cells that are highly sensitive for TNFR2-induced cell death^31^. Therefore, cells were incubated with the fusion proteins for 24 hours and cell viability was measured using the crystal violet assay. Again, bioactivity was increased with higher oligomerization of the TNF muteins (Fig. 5B and Table 3). No significant TNFR2 signal induction was observed in this cellular system for sc-mTNF_R2_ at the used concentrations. In contrast, p53-sc-mTNF_R2_ induced an approximately 10-times stronger signal than EHD2-sc-mTNF_R2_, whereas GCN4-sc-mTNF_R2_ was 10-times more potent than p53-sc-mTNF_R2_.

Next, we investigated TNFR2-induced proliferation of mouse thymocytes. Therefore, cells were incubated in presence of the different fusion proteins for 4 days and proliferation was determined by measuring their metabolic activity. All oligomerized fusion proteins induced proliferation. Whereas sc-mTNF_R2_ only promoted proliferation at higher concentrations, all oligomerized fusion proteins efficiently induced proliferation (Fig. 5C and Table 3). Whereas p53-sc-mTNF_R2_ (EC_50_ value 40 pM) was just slightly more potent than EHD2-sc-mTNF_R2_ (EC_50_ value 55 pM), GCN4-sc-mTNFR_2_ induced a more than 3-times increased response (EC_50_ value 16 pM) compared to EHD2-sc-mTNF_R2_.

**Figure 6:**
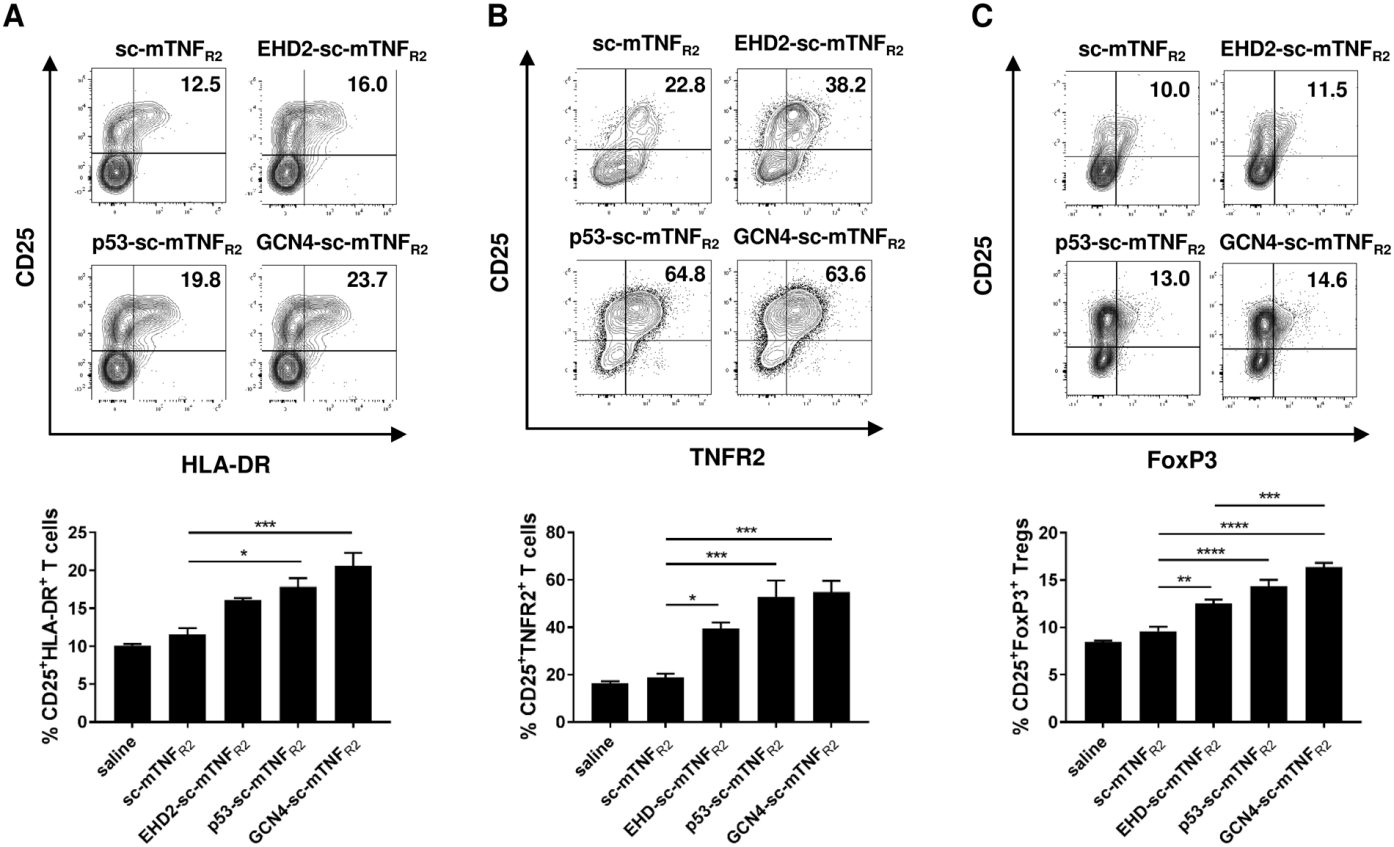
Dodecameric TNF muteins most potently induce T cell activation and Treg expansion (**A**) CD3^+^ T cells were isolated from human peripheral blood mononuclear cells (PBMCs) via magnetic separation. T cells were activated using plate-bound anti-CD3 (1 µg/ml) and cultivated in presence of interleukin 2 (IL-2, 10 U/ml) and 0.3 nM of the oligomerized TNF muteins for 4 days. Number of activated CD25^+^HLA-DR^+^ T cells was determined by flow cytometry. Shown is a representative donor (upper panel). Combined data are from three independent donors (n = 3 ± SEM, lower panel) (**B**,**C**) CD3^+^ T cells were isolated from mouse splenocytes. T cells were activated using plate-bound anti-CD3 (5 µg/ml) and cultivated in presence of interleukin 2 (IL-2, 100 U/ml) and 0.3 nM of the oligomerized TNF muteins for 4 days. Number of (**B**) activated CD25^+^TNFR2^+^ T cells and (**C**) CD25^+^FoxP3^+^ Tregs was determined by flow cytometry. Shown is a representative donor (lower panel). Combined data are from three independent experiments (n = 3 ± SEM, lower panel).

We next investigated TNFR2 signal induction in different immune cells (activated human and mouse T cells, regulatory T cells), known to express TNFR2^32–34^. We choose to use a concentration of 0.3 nM of the fusion proteins, due to the different effects observed in the previous assays for this concentration. Similar results were obtained for both, TNFR2-induced expansion of human CD25^+^HLA-DR^+^ (Fig. 6A and Table S1) and mouse CD25^+^TNFR2^+^ (Fig. 6B) activated T cells. Thus, GCN4-sc-mTNF_R2_ proved to be the strongest inducer of T cell proliferation, whereas no significant activity was recorded for sc-mTNF_R2_ compared to control cultures. For both, human and mouse T cells, p53-sc-mTNF_R2_ showed an apparent stronger activity than EHD2-sc-mTNF_R2_, although the difference was statistically not significant. Since TNFR2 is a key marker of regulatory T cells (Tregs)^32, 34^ and TNF is known to expand and stabilize Tregs via TNFR2^32, 35–37^, we then investigated the potential of the different sc-mTNF_R2_ fusion proteins to expand Tregs. Therefore, CD3^+^ mouse T cells were activated using αCD3, cultivated in presence of the tmTNF mimetics and the number of CD4^+^CD25^+^FoxP3^+^ Tregs was quantified by flow cytometry (Fig. 6C). Similarly to the previous experiments, the tetrameric GCN4-sc-mTNF_R2_ molecule was the most potent activator, inducing the strongest expansion of Tregs.

### Dodecavalent covalently stabilized scTNF show improved bioactivity *in vivo*

**Figure 7:**
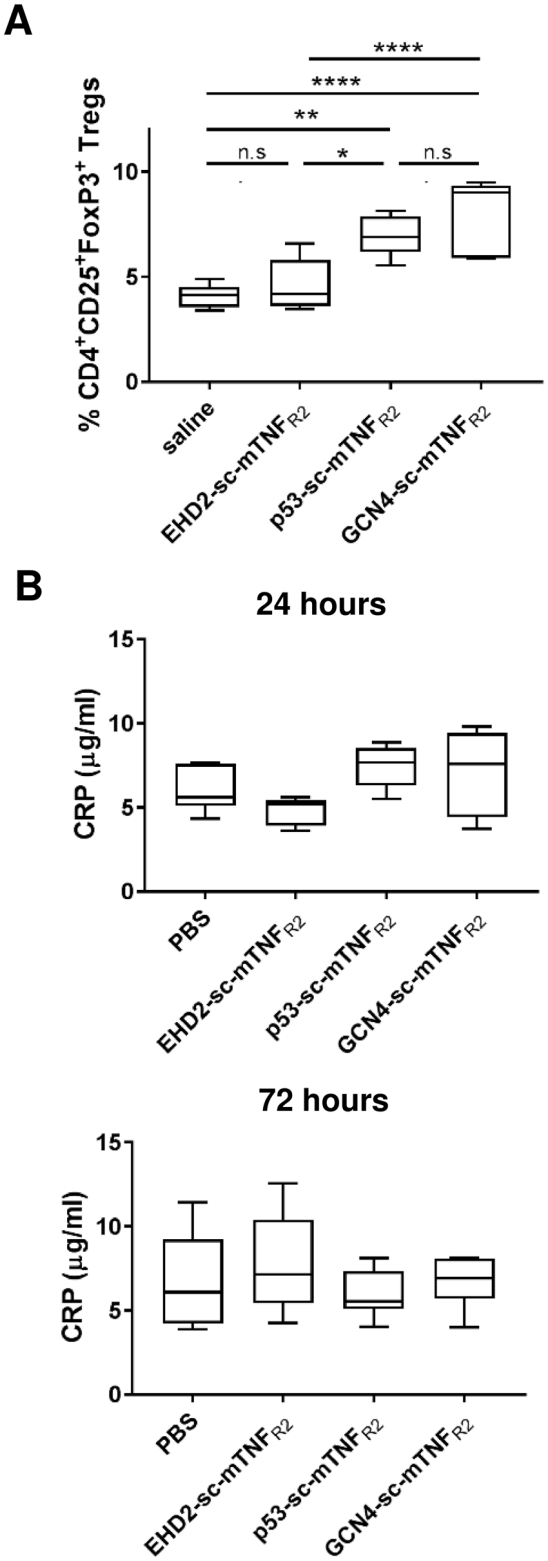
Dodecameric TNF muteins most potently induce T cell activation and Treg expansion C57BL/6 mice were administered with 1 mg/kg body weight (i.p.) of the proteins or saline. (**A**) After 3 days, splenocytes were isolated and number of CD4^+^CD25^+^FoxP3^+^ Tregs was determined by flow cytometry. (**B**) After 24 hours and 72 hours whole blood was withdrawn and CRP levels in the blood were determined by ELISA (n = 5–7 mice ± SEM).

Recently, it was shown that *in vivo* application of a TNFR2 agonist leads to the expansion of Tregs and protects mice from acute graft versus host disease^38^. Therefore, we administered low concentrations (1 mg/kg) of the TNF muteins EHD2-sc-mTNF_R2_, p53-sc-mTNF_R2_ and GCN4-sc-mTNF_R2_ in mice and determined the number of CD4^+^CD25^+^FoxP3^+^ Tregs in the spleen after three days (Fig. 7A). Interestingly no significant activity of EHD2-sc-mTNF_R2_ compared with saline control was observed. In contrast, both p53-sc-mTNF_R2_ and GCN4-sc-mTNF_R2_ efficiently expanded Tregs at the applied dose of 1 mg/kg *in vivo*. No significant difference was observed between p53-sc-mTNF_R2_ and GCN4-sc-mTNF_R2_ at this dose. However, at a tenfold higher concentration (10 mg/kg) EHD2-sc-mTNF_R2_ induced an efficient expansion of Tregs, too (Fig. S2). The systemic tolerance of the oligomeric TNF fusion proteins was assessed by measuring the levels of C-reactive protein (CRP), a key inflammatory marker, in the blood (Fig. 7B). Neither 24 nor 72 hours after administration of the TNF muteins, altered CRP levels compared to saline-treated animals were observed.

## Discussion

Several receptors of the TNF receptor superfamily depend on a transmembrane ligand to become robustly activated. Examples are the ligand/receptor pairs of CD95L and CD95, APRIL and TACI, OX40 L and OX40, TRAIL and TRAILR2, and TNF and TNFR2^14, 18, 39–41^. For mouse CD95L and TRAIL, soluble trimeric ligands solely containing the THD failed to bind to their cognate receptors and consequently were not able to activate receptor signaling. However, binding of these ligand variants to CD95 and TRAILR2 was restored by stabilization of the trimers via fusion to a trimerization domain. Although trimer stabilization restored receptor binding, this ligand still was not capable to efficiently activate its receptor^4^. Similar, covalent stabilization of TNF trimers improved the affinity to TNFR2, but did not improve the bioactivity^10^.

Previously, different approaches have been exploited to generate soluble TNF ligands mimicking the activity of the transmembrane form, e.g. by artificially increasing the avidity of TNF ligands through mutagenesis or by immobilizing them on a cell surface or the extracellular matrix (ECM). Antibody-mediated multimerization of Flag-tag-containing variants of soluble TNF, CD95L, TRAIL, and CD40L with Flag-specific antibodies resulted in a dramatic increase in avidity and efficient activation of their cognate receptors TNFR2, CD95, TRAILR2, and CD40, respectively^30, 42, 43^. Similar, antibody-mediated cross-linking of TNFR2 via the monoclonal non-activating antibody 80M2 leads to pre-activation of TNFR2 on the cell surface, thereby mimicking receptor activation by the membrane form of TNF, if soluble recombinant TNF is added^14^. Genetic fusion of soluble CD95L, OX40L trimers to an oligomerization domain resulted in a dramatic increase in avidity and efficiently activated CD95 and OX40^18, 30, 40^. Alternatively, immobilization of soluble ligands can elevate their activity. For instance, binding of soluble CD95L or soluble APRIL to components of the extracellular matrix converts these ligands into highly active cytokines^44–46^. Similar data show that the activity of soluble trimeric variants of CD27L, CD40L, and 41BBL can be strongly increased by oligomerization or cell surface immobilization^7^.

The dependence of the naturally occurring transmembrane ligand or membrane-mimetic variants for robust activation is especially obvious for TNFR2. Whereas it was shown that trimer stabilization both improves activation of TNFR1^10^ and TNFR2^47^, oligomerization of trimeric TNF ligands is highly superior in activating TNFR2^19–21^. In line with this, additional data indicate that mere mechanical fixation of TNF to an immobilized surface is insufficient to efficiently activate TNFR2. Rather, a certain density of the ligand and an additional stabilization of TNFR2 by cluster formation seems to be mandatory for efficient activation^48^. Whereas data exist that oligomerization of a trimeric scTNF ligand improves its bioactivity, no data were available on the possible stoichiometry and composition of oligomerized TNF ligands necessary to robustly activate TNFR2.

Receptor binding studies revealed that the dodecavalent variants p53-sc-mTNF_R2_ and GCN4-sc-mTNF_R2_ both bind more stable to TNFR2 compared to the hexavalent EHD2-sc-mTNF_R2_. Furthermore, using different cellular response assays, GCN4-sc-mTNF_R2_ consistently exerted the highest bioactivity. In addition, in all assays a slightly increased bioactivity of p53-sc-mTNF_R2_ compared to EHD2-sc-mTNF_R2_ was observed. A first important step for efficient signal induction is the formation of higher order TNF/TNFR2 signaling clusters. Our microscopical analyses showed that GCN4-sc-mTNF_R2_ efficiently promoted the cluster formation even at low sub-nanomolar concentrations. Likewise, p53-sc-mTNF_R2_ was superior in cluster-formation compared to EHD2-sc-mTNF_R2_, which translated into a 3.5-fold higher bioactivity in a Cxcl-2 chemokine secretion assay with BV-2 cells.

Altogether, our data show that the three-dimensional orientation of tmTNF-mimetic TNF ligands is important and determines the efficiency of TNFR2 signal induction. Our data suggest that the configuration of GCN4-sc-mTNF_R2_ shares the highest similarity with the assumed natural tmTNF configuration, since all TNF trimers in the oligomer are arranged in a way that they point into the same direction. In contrast, p53-sc-mTNF_R2_ seems to have more a spider-like shape in which the binding sites point into the directions of a tetrahedron^24^. We, therefore, hypothesize that these different 3D-structures mainly impact the distance between the scTNF units, with the highly potent GCN4-sc-mTNF_R2_ configuration displaying the least distance between the scTNF units. Therefore, it is likely that the distance between TNF trimers in tmTNF or tmTNF mimetic ligands also plays a role for the bioactivity. Interestingly, the *in vivo* application of the tetramers p53-sc-mTNF_R2_ and GCN4-sc-mTNF_R2_ is highly superior to dimeric EHD2-sc-mTNF_R2_, whereas no significant differences between p53-sc-mTNF_R2_ and GCN4-sc-mTNF_R2_ were observed. This superior bioactivity of p53-sc-mTNF_R2_ *in vivo* compared to the *in vitro* assays might be due to possible *cis* and *trans* activation of TNFR2 on the same and on neighboring cells, respectively. We propose that a p53-sc-mTNF_R2_ molecule that binds to TNFR2s with a minimum of two scTNFs is capable of activating TNFR2, similar to EHD2-sc-mTNF_R2_, but still possesses two free scTNFs capable of activating TNFR2 *in trans* on other cells. Keeping in mind that TNF/TNFR2 clusters are formed, such a higher order p53-sc-mTNF_R2_/TNFR2 cluster with free scTNF molecules could mimic natural tmTNF signaling, i.e. an additional transactivation of a juxtaposed cell.

Since TNFR2 plays an important role in immune regulation and tissue regeneration, ligands that effectively activate TNFR2 hold great promise as novel therapeutics to treat a variety of diseases, including inflammatory and neurodegenerative diseases^11, 13, 49, 50^. Drug potency and efficacy is of particular interest for therapeutic applications, as lower doses of the particular drug are needed to induce maximal therapeutic effects. Therefore the high and specific bioactivity of tmTNF-mimetic tetrameric scTNF oligomers represent a novel approach towards TNFR2-selective drugs.

In conclusion, we show that controlled oligomerization of scTNFs is a prerequisite to efficiently activate TNFR2. By introducing various oligomerization domains to generate dimeric or tetrameric scTNF ligands with different three-dimensional orientation of the scTNF units, we identified formats suitable for efficient TNF/TNFR2 complex formation and signal induction and high bioactivity in *in vitro* and *in vivo* model systems. Our findings, therefore, are of principal relevance for development of TNFR2 agonists for potential therapeutic application. For the use in humans completely human molecules are needed. This can be readily achieved by exchanging the sc-mTNF_R2_ part to its human counterpart scTNF_R2_ which was previously described by our group^19, 21^. The yeast GCN4 tetramerization domain was used here as a model of a coiled-coil structure. To circumvent potential immunogenicity of foreign protein domains, this domain could be exchanged to similar coiled-coil tetramerization domains of human proteins. In addition, other modules and formats resulting in a GCN4-sc-mTNF_R2_-like configuration are possible to design highly potent TNFR2 agonists.

In summary, our data show that TNFR2 activation by dodecavalent ligands is clearly superior to trivalent or hexavalent ligands. We also demonstrate that the predicted 3D-structure of a dodecavalent TNFR2-selective TNF mutein appears to be important for the bioactivity of the ligand. In addition, because of conserved structural features of the members of the TNF/TNFR superfamily, our findings suggest that similar conditions exist for other members of this biologically important cytokine family.

## Materials and Methods

### Materials

The TNFR2-specific antibody 80M2 has been described^14^. The anti-TNF antibody (clone F6C5) was from GeneTex (Irvine, CA) and anti-TNF (HP8001) from Hycult Biotech (Uden, The Netherlands). The anti-CD3 (clone UCHT1 & 17A2) and anti-TNFR2 antibody (AF-426-PB) were from R&D Systems (Wiesbaden-Nordenstadt, Germany). Fluorescence-labeled antibodies against CD3, CD4, CD25, HLA-DR, TNFR2 and FoxP3 were from Miltenyi Biotech (Bergisch-Gladbach, Germany). Secondary antibodies coupled to Alexa Fluor 488 or Alexa Fluor 546 were from Life Technologies (Karlsruhe, Germany) and horseradish peroxidase (HRP)-labeled anti-mouse IgG antibodies were purchased by Jackson ImmunoResearch Laboratories (Suffolk, UK). Recombinant interleukin 2 (IL-2) was purchased by Immunotools (Friesoythe, Germany). Actinomycin D, 4′,6-Diamidin-2-phenylindol (DAPI) and 3-(4,5-dimethyl-2-thiazolyl)−2,5-diphenyl-2H-tetrazolium bromide (MTT) were from Sigma-Aldrich and 3,30,5,50-tetramethylbenzidine (TMB) substrate was purchased from Biolegend (San Diego, CA). All other chemicals were of analytical grade.

### Production and purification of TNFR2 agonists

Production and purification of recombinant proteins was described previously^21^. Briefly, HEK293-6E cells, grown in F17 medium (Life Technologies, Darmstadt, Germany), were transiently transfected with expression constructs of TNF muteins using polyethyleneimine (Sigma). The day after, Tryptone N1 (Organotechnie, TekniScience, Terrebonne, Canada) was added to the cell culture and cells were cultivated for additional 4 days. Then, supernatant was collected and recombinant proteins were purified by immobilized metal ion chromatography (IMAC). For this purpose, supernatant was loaded onto a column containing Ni-NTA agarose (Macherey-Nagel, Düren, Germany) and unbound proteins were washed away using IMAC wash buffer (50 mM sodium-phosphate-buffer). Bound proteins were eluted with IMAC elution buffer (50 mM sodium-phosphate-buffer, 250 mM imidazole) and dialyzed (cut-off 4–6 kDa, Roth, Karlsruhe, Germany) against PBS overnight at 4°C. Finally, eluted proteins were purified by SEC. Protein concentration was determined by measuring the absorbance at 280 nm. For coomassie staining, 2 µg of the purified TNF muteins were denatured in Laemmli buffer, resolved by 8% SDS-PAGE and stained with coomassie.

### HPLC

Approx. 20 µg protein was applied to a BioSep-SEC-S2000 column (Phenomenex, Aschaffenburg, Germany) equilibrated with PBS and eluted at a flow rate of 0.5 ml/min. For determining the size of recombinant proteins, standard proteins were run under the same conditions.

### TNFR binding assay

ELISA plates (Greiner, Frickenhausen, Germany) were coated with TNFR1-Fc or TNFR2-Fc fusion proteins at 1 µg/ml in PBS and incubated at 4°C overnight. Residual binding sites were blocked with 2% skim milk powder in PBS at RT for 2 hours. TNF muteins were diluted in 2% skim milk powder in PBS and incubated for 1 hour at RT. Bound proteins were detected with mouse monoclonal antibodies to TNF (clone F6C5; 1 µg/ml; incubation for 1 hour at RT) and HRP-conjugated anti-mouse IgG antibodies (diluted 1:10000; incubation for 1 hour at RT), followed by incubation with TMB substrate solution. Reaction was stopped by addition of 1 M H_2_SO_4_ and the absorbance at 450 nm was determined with an absorbance reader (Multiskan FC, Thermo Scientific, Karlsruhe, Germany) and data were analyzed using the software Microsoft Excel and GraphPad Prism 4 (GraphPad, La Jolla, CA). Between each step, non-bound proteins were removed by washing 4 times with 0.005% Tween-20 in PBS.

### Attana

Affinities of the oligomerized fusion proteins for TNFR2-Fc were determined by quartz crystal microbalance measurements (Attana Cell 200, Attana, Stockholm, Sweden). Therefore, TNFR2-Fc fusion proteins were chemically immobilized on a carboxyl sensor chip according to the manufacturer’s protocol at a high (270 Hz) and low (130 Hz) density, respectively. Binding experiments were performed in PBST (PBS, 0.1% Tween 20) pH 7.4 at a flow rate of 25 ml/min at 37 °C. The chip was regenerated with 10 mM Glycine HCl pH 2.0. Before each measurement, a baseline was measured which was subtracted from the binding curve. Data were collected using Attaché Office software (Attana, Stockholm, Sweden) and TraceDrawer (Ridgview Instruments, Vange, Sweden).

### Cytotoxicity assay

L929 or Kym-1 cells (1.5 × 10^4^ cells/well) were grown in 96-well flat bottom cell culture plates overnight. L929 cells were treated with actinomycin D (1 µg/ml) and Kym-1 cells with 80M2 (1 µg/ml) for 30 minutes prior to addition of TNF muteins. L929 and Kym-1 cells were incubated with different concentrations of TNF muteins for 24 hours at 37 °C. Cells were washed with PBS and incubated with crystal violet (20% methanol; 0.5% crystal violet) for 20 minutes to stain viable cells. The dye was washed away under rinsing water and cells were air-dried. Crystal violet was resolved with methanol and the optical density at 550 nm was determined. Each sample was analyzed in triplicates and data were analyzed using the software Microsoft Excel and GraphPad Prism (GraphPad, La Jolla, CA).

### Enzyme-linked immunosorbent assay

BV-2 or HeLa cells were stimulated as indicated, supernatants were collected after 24 hours and analyzed by an ELISA specific for Cxcl-2 (BV-2, R&D Systems, Minneapolis MN) or IL-6 (HeLa, Biolegend, San Diego CA) according to the instructions of the manufacturer. For determination of inflammatory marker CRP in the blood, whole blood was withdrawn and analyzed by an ELISA specific for mouse CRP (R&D Systems, Minneapolis MN) according to the instructions of the manufacturer. The absorbance at 450 nm was determined and the amount of released Cxcl-2 or IL-6 was determined with the provided standard and calculated using the software GraphPad Prism.

### Thymocyte proliferation

Thymus of C57BL/6 mice was isolated and mashed through a 40 µm cell strainer (Flacon). Cells were centrifuged (300 *g*, 5 min) and washed once with culture medium (RPMI 1640, 10% FCS, 50 µM β-mercaptoethanol and penicillin/streptomycin). Then 1.5 × 10^5^ cells were plated into αCD3 coated (6 h at 4 °C, 1 µg/ml) 96-well (U) plates and cultivated for 4 days in presence of the oligomerized fusion proteins. Number of cells was determined by MTT (3-(4,5-Dimethylthiazol-2-yl)−2,5-Diphenyltetrazolium Bromide) assay. Therefore, cells were incubated with MTT (0.5 mg/ml) for 2 hours at 37 °C. Then lysis buffer (10% SDS, 20 nM HCl) was added, cells were lyzed overnight and optical density at 550 nm was determined. Each sample was analyzed in triplicates and data were analyzed using the software Microsoft Excel and GraphPad Prism.

### Biological samples

Blood was obtained from volunteer donors, from whom informed consent was obtained. Blood withdrawal was carried out in accordance with relevant guidelines and regulations and all experimental protocols were approved by Ethik-Kommision Universitätsklinikum Tübingen (Project number 283/2014B02).

### Human T cell isolation and culture

Blood of volunteer human donors was diluted 1:2 with RPMI medium. Then 30 ml diluted blood was layered over 10 ml Histopaque-1077 (Sigma-Aldrich) and centrifuged for 20 min at 800 *g* without brake. Interphase, including mononuclear cells of peripheral blood (PBMCs) was removed and washed with 30 ml RPMI (300 *g*, 5 min). For removal of platelets, cells were resuspended in 40 ml RPMI and centrifuged for 5 min at 200 *g*. Then, CD3^+^ T cells were isolated by magnetic separation using the Pan T Cell Isolation Kit (Miltenyi Biotech). Purified T cells were plated in X-Vivo 15 medium (Lonza) in αCD3 coated (6 h at 4 °C) 96-well (U form) plates for T cell activation. Cells were incubated in presence of IL-2 and the oligomerized fusion proteins for 4 days. Then surface expression of CD25 and HLA-DR was determined by flow cytometry according to manufacturer’s instructions (Miltenyi Biotech, Bergisch-Gladbach). At least 20,000 cells per sample were acquired. Data were acquired using a MACSQuant Analyzer 10 (Miltenyi) and analyzed with FlowJo (FlowJo, LLC, Ashland, Oregon).

### Mouse T cell isolation and culture

Spleens from C57BL/6 wildtype mice were dissociated through a 40 µm cell strainer and collected in 10 ml MACS buffer (PBS, 0.5% BSA, 2 mM EDTA). Splenocytes were centrifuged (300 *g*, 5 min) and washed once with 10 ml MACS buffer. Then CD3^+^ T cells were isolated using the FACS Aria III and plated in αCD3 coated (6 h at 4°C) 96-well (U form) plates for T cell activation. T cells were cultivated in presence of IL-2 and the oligomerized fusion proteins for 4 days. Then expression of CD25, TNFR2 and/or FoxP3 was determined by flow cytometry according to manufacturer’s instructions (Miltenyi Biotech, Bergisch-Gladbach). Data were acquired using a MACSQuant Analyzer 10 (Miltenyi) and analyzed with FlowJo (FlowJo, LLC).

### *In vivo* Treg assay

Animal care and treatment were carried out in accordance with Committee Stuttgart (Regierungspräsidium Stuttgart) guidelines on the use of experimental animals at the University of Stuttgart. Animal experiments were approved by Regierungspräsidium Stuttgart (permit no. 35-9185.81/0422). Oligomerized fusion proteins (1 mg/kg) were administered intraperitoneal (i.p.) to C57BL/6 wild type mice. After 3 days a second injection (1 mg/kg) was applied. After 7 days, spleens were extracted and splenocytes were isolated. Therefore, spleens were dissociated through a 40 µm cell strainer and collected in 10 ml MACS buffer (PBS, 0.5% BSA, 2 mM EDTA). Splenocytes were centrifuged (300 *g*, 5 min) and incubated in 3 ml RBC buffer (0.15 M NH_4_Cl, 10 mM KHCO_3_, 0.1 M EDTA) per spleen for 5 minutes at room temperature to lyse red blood cells. Then 10 ml MACS buffer was added and splenocytes were centrifuged for 5 min at 300 *g*. Cells were washed once with 10 ml MACS buffer (5 min, 300 *g*) and afterwards collected in MACS buffer. Expression of markers CD25 and FoxP3, within the subpopulation of CD4^+^ T cells, was determined by flow cytometry according to manufacturer’s instructions (Miltenyi Biotech, Bergisch-Gladbach). Data were acquired using a MACSQuant Analyzer 10 (Miltenyi) and analyzed with FlowJo (FlowJo, LLC).

### Quantification of TNF/TNFR2 complex clustering

BV-2 cells were stimulated with TNF muteins for 15 minutes at 37 °C. Then cells were immediately washed two times with ice cold PBS and fixed with 4% PFA in PBS solution. Then unspecific binding sites were blocked with 4% BSA in PBS and cells were incubated with antibodies against TNF (HP8001, Hbt) and TNFR2 (AF-426-PB, R&D systems), followed by detection with appropriate fluorescence labeled antibodies. Nuclei were counterstained with DAPI. Stainings were analyzed on a Zeiss Axio Observer Spinning Disc microscope equipped with a Plan-Apochromat 63x/1.4 Oil DIC objective and an Axiocam 503 mono CCD camera. The following excitation lasers and emission filters were used: DAPI: 405 diode laser, 450/50 nm filter; GFP, 488 nm diode laser, 525/50 nm filter; RFP, 561 nm (RFP) diode laser, 600/50 nm filter. Z-stacks of tile regions containing 6 × 6 images were acquired and maximum intensity projections were calculated. Image processing was done in Zen blue 2.1 software (Zeiss, Germany).

Quantitative image analysis was done with CellProfiler version 2.2^51^. Nuclei were segmented via the DAPI staining and a 120 pixel wide ring mask was drawn around each nucleus representing the cell mask. TNF Vesicles were segmented under the cell mask using the A488 staining and unified for each cell. Mean intensity of the TNFR2 signal using the A546 staining was measured under the unified vesicles representing the grade of co-localized vesicles between TNFR2 (red) and TNF (green) per cell.

### Statistics

Data are presented as mean ± standard error of the mean (SEM) of *n* independent experiments. Normal distribution was analyzed by Shapiro-Wilk normally test. Statistical analyses were performed by Student’s t-test or analysis of variance, followed by a post hoc Tukey’s range test. *P < 0.05 (**P < 0.01; ***P < 0.001; ****P < 0.0001) was considered significant.

## Electronic supplementary material

Supplementary Data
