# Supplementary material for: Novel strategies to mimic transmembrane tumor necrosis factor-dependent activation of tumor necrosis factor receptor 2
Source: Sci Rep. 2017 Jul 26;7:6607. doi: 10.1038/s41598-017-06993-4 (PMC5529482; doi:10.1038/s41598-017-06993-4)
Supplement: Supplementary file 1 — Supplementary Data [file 41598_2017_6993_MOESM1_ESM.doc]

Novel strategies to mimic transmembrane tumor necrosis factor-dependent activation of tumor necrosis factor receptor 2

**Authors:** Roman Fischer^1,2*^, Jessica Marsal^1^, Cristiano Guttà^1^, Stephan A. Eisler^3^, Nathalie Peters^1^, John R. Bethea^2^, Klaus Pfizenmaier^1^ and Roland E. Kontermann^1*^

**sc-mTNF_R2_**

SDKPVAHVVANHQVEEQLEWLSQRANALLANGMDLKDNQLVVPADGLYLVYSQVLFKGQGCPDYVLLTHTVSRFAISYQEKVNLLSAVKSPCPKDTPEGAELKPWYEPIYLGGVFQLEKGDQLSAEVNLPKYLNFRESGQVYFGVIALGGGGSSSDKPVAHVVANHQVEEQLEWLSQRANALLANGMDLKDNQLVVPADGLYLVYSQVLFKGQGCPDYVLLTHTVSRFAISYQEKVNLLSAVKSPCPKDTPEGAELKPWYEPIYLGGVFQLEKGDQLSAEVNLPKYLNFRESGQVYFGVIALGGGGSSSDKPVAHVVANHQVEEQLEWLSQRANALLANGMDLKDNQLVVPADGLYLVYSQVLFKGQGCPDYVLLTHTVSRFAISYQEKVNLLSAVKSPCPKDTPEGAELKPWYEPIYLGGVFQLEKGDQLSAEVNLPKYLNFRESGQVYFGVIAL

**EHD2-sc-mTNF_R2_**

METDTLLLWVLLLWVPGSTGDAAQPAGGGAAAHHHHHHGGTGGGGSGGKLGGSGGDFTPPTVKILQSSCDGGGHFPPTIQLLCLVSGYTPGTINITWLEDGQVMDVDLSTASTTQEGELASTQSELTLSQKHWLSDRTYTCQVTYQGHTFEDSTKKCADSNGGGSGGGSGGGSGGGSGGGSGGSGIRSDKPVAHVVANHQVEEQLEWLSQRANALLANGMDLKDNQLVVPADGLYLVYSQVLFKGQGCPDYVLLTHTVSRFAISYQEKVNLLSAVKSPCPKDTPEGAELKPWYEPIYLGGVFQLEKGDQLSAEVNLPKYLNFRESGQVYFGVIALGGGGSSSDKPVAHVVANHQVEEQLEWLSQRANALLANGMDLKDNQLVVPADGLYLVYSQVLFKGQGCPDYVLLTHTVSRFAISYQEKVNLLSAVKSPCPKDTPEGAELKPWYEPIYLGGVFQLEKGDQLSAEVNLPKYLNFRESGQVYFGVIALGGGGSSSDKPVAHVVANHQVEEQLEWLSQRANALLANGMDLKDNQLVVPADGLYLVYSQVLFKGQGCPDYVLLTHTVSRFAISYQEKVNLLSAVKSPCPKDTPEGAELKPWYEPIYLGGVFQLEKGDQLSAEVNLPKYLNFRESGQVYFGVIAL

**p53-sc-mTNF_R2_**

METDTLLLWVLLLWVPGSTGDAAQPAGGGAAAHHHHHHGGTGGGGSGGKLKKPLDGEYFTLQIRGRERFEMFRELNEALELKDAQAGKEPGAPGGGSGGGSGGGSGGGSGGGSGGSGIRSDKPVAHVVANHQVEEQLEWLSQRANALLANGMDLKDNQLVVPADGLYLVYSQVLFKGQGCPDYVLLTHTVSRFAISYQEKVNLLSAVKSPCPKDTPEGAELKPWYEPIYLGGVFQLEKGDQLSAEVNLPKYLNFRESGQVYFGVIALGGGGSSSDKPVAHVVANHQVEEQLEWLSQRANALLANGMDLKDNQLVVPADGLYLVYSQVLFKGQGCPDYVLLTHTVSRFAISYQEKVNLLSAVKSPCPKDTPEGAELKPWYEPIYLGGVFQLEKGDQLSAEVNLPKYLNFRESGQVYFGVIALGGGGSSSDKPVAHVVANHQVEEQLEWLSQRANALLANGMDLKDNQLVVPADGLYLVYSQVLFKGQGCPDYVLLTHTVSRFAISYQEKVNLLSAVKSPCPKDTPEGAELKPWYEPIYLGGVFQLEKGDQLSAEVNLPKYLNFRESGQVYFGVIAL

**GCN4-sc-mTNF_R2_**

METDTLLLWVLLLWVPGSTGDAAQPAGGGAAAHHHHHHGGTGGGGSGGKLRLKQIEDKLEEILSKLYHIENELARIKKLLGERGAPGGGSGGGSGGGSGGGSGGGSGGSGIRSDKPVAHVVANHQVEEQLEWLSQRANALLANGMDLKDNQLVVPADGLYLVYSQVLFKGQGCPDYVLLTHTVSRFAISYQEKVNLLSAVKSPCPKDTPEGAELKPWYEPIYLGGVFQLEKGDQLSAEVNLPKYLNFRESGQVYFGVIALGGGGSSSDKPVAHVVANHQVEEQLEWLSQRANALLANGMDLKDNQLVVPADGLYLVYSQVLFKGQGCPDYVLLTHTVSRFAISYQEKVNLLSAVKSPCPKDTPEGAELKPWYEPIYLGGVFQLEKGDQLSAEVNLPKYLNFRESGQVYFGVIALGGGGSSSDKPVAHVVANHQVEEQLEWLSQRANALLANGMDLKDNQLVVPADGLYLVYSQVLFKGQGCPDYVLLTHTVSRFAISYQEKVNLLSAVKSPCPKDTPEGAELKPWYEPIYLGGVFQLEKGDQLSAEVNLPKYLNFRESGQVYFGVIAL

**Supplementary Figure 1 Amino acid sequence of the fusion proteins.**


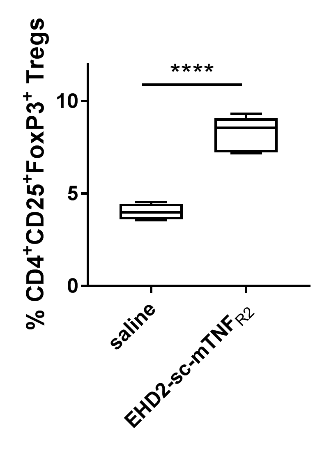


**Supplementary Figure 2 TNFR2-dependent Treg expansion** C57BL/6 mice were administered with 10 mg/kg body weight (i.p.) of EHD2-sc-mTNF_R2_ or saline. (A) After 3 days, splenocytes were isolated and number of CD4^+^CD25^+^FoxP3^+^ Tregs was determined by flow cytometry. (B) After 24 hours and 72 hours whole blood was withdrawn and CRP levels in the blood were determined by ELISA (n=5-7 mice ±SEM).

**Table S1** Percentage of CD3^+^CD25^+^HLA-DR^+^ T cells for 3 independent donors

|  | **saline** | **sc-mTNF_R2_** | **EHD2-sc-mTNF_R2_** | **p53-sc-mTNF_R2_** | **GCN4-sc-mTNF_R2_** |
| --- | --- | --- | --- | --- | --- |
| **Donor #1** | 9.89 | 9.94 | 15.6 | 15.8 | 17.6 |
| **Donor #2** | 10.53333 | 12.26667 | 16.6 | 17.93333 | 20.4 |
| **Donor #3** | 9.8 | 12.5 | 16 | 19.8 | 23.7 |
